# Normative values for hand grip and pinch strength for 6 to 18 year-olds in Saudi Arabia

**DOI:** 10.1186/s12891-023-06197-0

**Published:** 2023-02-06

**Authors:** Bader A. Alqahtani, Aqeel M. Alenazi, Ragab K. Elnaggar, Mohammed M. Alshehri, Ahmed Alhowimel, Ayat A. Najmi, Muneera Alasraj, Mshari Alghadeir

**Affiliations:** 1 Department of Health and Rehabilitation Sciences, Prince Sattam Bin Abdulaziz University, Al-Kharj, 11942 Kingdom of Saudi Arabia; 2 Department of Physical Therapy and Health Rehabilitation, Prince Sattam Bin Abdulaziz University, Al-Kharj, 11942 Kingdom of Saudi Arabia; 3 Department of Physical Therapy for Pediatrics, Faculty of Physical Therapy, Cairo University, Giza, Egypt; 4 Physical Therapy Department, Jazan University, Jazan, Kingdom of Saudi Arabia

**Keywords:** Pinch, Grip, Handheld dynamometer, Normative, Strength

## Abstract

**Background:**

Normative values for hand grip and pinch strength among children in Saudi Arabia has not been well established. Therefore, the main aim of this study is to establish normative values for hand grip and pinch strength in children aged 6 to 18 years in Saudi Arabia.

**Methods:**

A cross-sectional study was conducted from different 5 regions in Saudi Arabia. Participants between the age of 6 years and 18 years old were recruited through different primary and secondary schools in Saudi Arabia. Data for age, gender, Body Mass Index, and preferred hand were collected. Hand grip strength was measured using digital hand dynamometer and the tip pinch, palmar pinch, and key pinch strength were measured using the hydraulic pinch gauge.

**Results:**

A total of 616 participants included in this study (318 boys and 298 girls). Participants were stratified into 5 chronological age groups of 6–7 years, 8–9, 10–11, 12–13, 14–15, 16–17, and 18 years. The results showed an overall trend of increasing hand grip strength and pinch strength with age regardless of hand preference. Boys had significantly higher grip strength than girls in all age groups (*P* < 0.05).

**Conclusion:**

This study established normative values for hand grip and pinch strength in the healthy Saudi pediatric and adolescent population, using boys and girls aged 6 to 18. The outcomes of this study also demonstrated that gender, age, and hand preference can all have an impact on how strong a handgrip develops.

## Introduction

The hand is the upper limb’s most dynamic and collaborative part. The complex structure and function of the hands converge primarily in the hand gripping, one that is observed constantly during activities of daily living (ADLs). Accordingly, grip and pinch strength analysis is a key component of upper limb functional evaluation [1].

There are currently no published normative data on grip and pinch strength in Saudi Arabia that were obtained from a single sample of children between the ages of 6 and 18 and stratified by age at 2-year intervals. In addition, much of the data were not collected in accordance with the standard procedures that ensure valid and reliable measurements [2].

Grip strength is a common clinical metric that is consistently used to evaluate physical abilities in adults and children [3]. It primarily represents the maximum force produced by the simultaneous contraction of the hand and forehand muscles involved in the handgrip performance [4]. Measurements of grip strength have frequently been employed in various contexts as a functional evaluation of the overall strength and has also been used in epidemiological and experimental investigations of children and adolescents [4, 5]. Pinch strength refers to the type of prehension in which two or three fingers are used in synchronization with thumb movements for managing objects without interaction with the palm [6, 7]. Tip pinch, key pinch, and palmar pinch are the three main pinch strength measurements that have been evaluated in clinical settings [8].

The measures of hand grip and pinch strength are commonly used in clinical practice as an objective indicators of upper limb functionality [9] and are influenced by many factors, including age, gender, race/ethnicity, body mass index, level of physical activity, and comorbidities [10, 11]. Another key factor in determining the hand grip and pinch strength is the anthropometric dimensions of the hand (length and width) [12].

Since the grip and pinch strength and associated factor have become a major public health concern, particularly among children, thus, there may be a rationale for further research to establish national normative data of grip and pinch strength and explore the associated factors.

While also grip and pinch strength are commonly used in clinical practice as objective indicators of upper limb functionality [9], it might be essential to collect current and comprehensive nationally representative data using rigorous procedures in order to make informed decisions about assessment and intervention. More specifically, normative values established in the present study for children aged from six to 18 years could be referenced for determining the individual’s degree of disability, establishing clinical, surgical, or rehabilitation goals, verifying the treatment efficacy through the patient’s evolution, and assessing the functional prognosis [13].

Over the last few years, several studies which provide normative data for hand grip and pinch grip strength in children of various age ranges have been published on both an international and domestic level. Normative data were collected internationally using hand-held devices, most commonly for grip strength [14–18], and less frequently for pinch strength [1, 19, 20]. A rigorous review of these studies implied a reduction in grip strength over the last three decades and concluded that hand function (i.e., hand grip and pinch grip strength) may change across generations and countries over time [13–15, 21].

Data from Spain, which was collected from data collected from 2001 to 2002 and from 2006 to 2007 revealed a 4.5 kg decrease in hand strength in adolescents of both genders between the ages of 12.5 and 17.5 years [16]. Records from England showed a 6.3% reduction in hand grip strength, which was collected from 10-years-old children over the period from 1998 to 2009 [17]. Stats from Canada indicated that between 1981 and 2009 for children aged 6-19 years, the hand grip strength decreased by 5 kg in a typical 12-year-old boy and 4 kg in a typical 12-year-old girl [18]. A study by Butterfield concluded that hand grip strength was increased with age and male presented with higher hand grip strength compared to female, however the hand dominance was not associated with the increased in hand grip strength [22]. In a recent study published in Saudi Arabia on female colleage students aged 19 to 25 years found that hand grip strength increased with age and dominant hand showed greater strength compared to nondominant hand [23]. In a study with Iranian children and adolescnts, a hand grip strength increased lineay for boys and girls until the age of 11 years however boys shoed steeper increase compared to girls [24]. Pinch grip strength was greater in Indian male compared to female counterparts and it was associated with increase in age [25].

The national studies providing normative values for grip and pinch strength in Saudi Arabia are limited by small sample size or a sampling bias due to inclusion of children in a narrow age range, single-site recruitment, and lack of representatives of different socio-cultural and economic aspects, making their data only generalizable to a narrow population [11, 21, 26]. Besides, the majority of Western countries’ normative data on the hand grip and pinch strength might not be applicable to Saudi children considering differences in demographic/physical characteristics, race, ethnicity, and lifestyle. Thus establishing normative data of hand grip and pinch strength for each population and region is important to assess hand impainrment and improve clinical management.

Therefore, the present study was conducted to establish normative values stratified by age and gender-related differences for hand grip strength and pinch strength in healthy Saudi children in the 6- to18-years-old range and to explore their relationship with specific demographic and anthropometric measurements.

## Methods

### Study design and sample

**Fig. 1:**
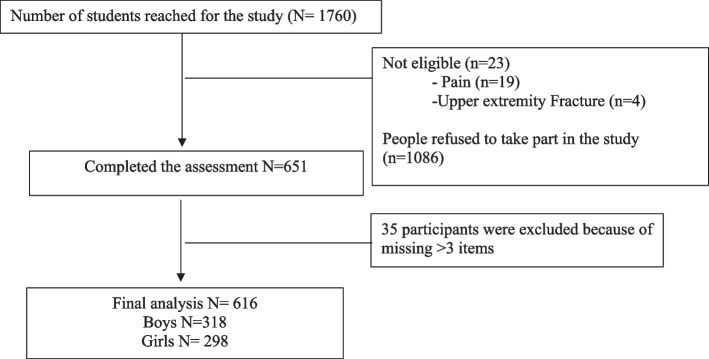
Flow chart of participants’ enrollment

A community-based cross-sectional study was conducted in different cities in Saudi Arabia, included a total of 616 participants (318 Boys) (Fig. 1). Participants were recruited through different primary and secondary schools in Riyadh and Jazan regions in Saudi Arabia, central and southern regions. Participants with any neurological dysfunctions, upper limb pain and deformity, functional limitation, impaired cognition, or any condition that prevents them from task execution were excluded. All participants and their parents signed a consent form describing the aims of the study and its procedure. The study was approved by the Ethical Committee of Prince Sattam bin Abdulaziz University in Saudi Arabia (5/112020).

### Instrument and procedure

Demographic data including age, gender, and preferred hand were collected using a questionnaire. Outcome meaures have been collected by a trained physical therapists. The preferred hand was determined according to Peter (1998) [19], with an observation of performance of activity of daily living such as writing, reaching, and grasping. The data collection followed the American Society of Hand Therapists (ASHT) recommendations [20]. Each participant sat on a chair facing the examiner with feet flat on the floor, shoulders adducted, neutrally rotated, elbow flexed at 90°, forearm in a neutral position, and wrist placed between 0° and 30° extension and between 0° and 15° ulnar deviation. A verbal instructions and test position demonstration were provided, followed by practice session that included three practice trials alternating the preferred and non-preferred hand. Hand grip strength (HGS) was measured using GRIPX Digital Hand Dynamometer (GRIPX, China) and the tip pinch, palmar pinch, and key pinch strength were measured using the Baseline Hydraulic Pinch Gauge (Fabrication Enterprises, Inc., USA). Participants were instructed to squeeze as hard as possible and to maintain the position for 5 seconds. Participants performed three trials for each hand (specified by dominant and non-dominant hand), the mean values of these trials were recorded. To minimize the effect of fatigue 1 min of rest was provided between each trial [21].

### Data analysis

Data was analyzed using statistical software Stata version 15.1 (Stata Corp, College Station, TX). Descriptive data for the current sample was stratified by gender and dominance hand. Freqcuency and percentages were calculated for categorical data. Means and standard deviations were calculated for continlus data. Normality of the included variables was assessed using Kolmogorov-Smirnov test. For the comparison of grip and pinch strength between boys and girls, U Mann-Whitney test was utilized. A *p*-value <.05 was considered significant. The traditional selection of .05 is really an arbitrary in the literature. Depending on how critical a Type I error, a researcher can select other criterion levels such as 0.01 or 0.1. For example, when we use alpha = .05 as our criterion for rejecting the null hypothesis (H0), we would have in this case only 5 out of 100 chances of making a Type I error. This indicates that we could have greater confidence in our decision to reject the null hypothesis. Type I error indicates mistakenly finding a difference while actually there is no difference.

## Results

**Fig. 2:**
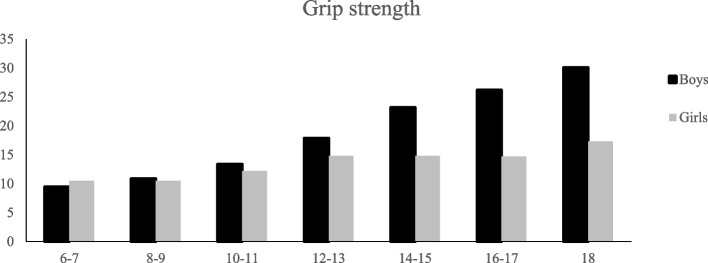
Average Performance of girls and boys on grip strength

**Fig. 3:**
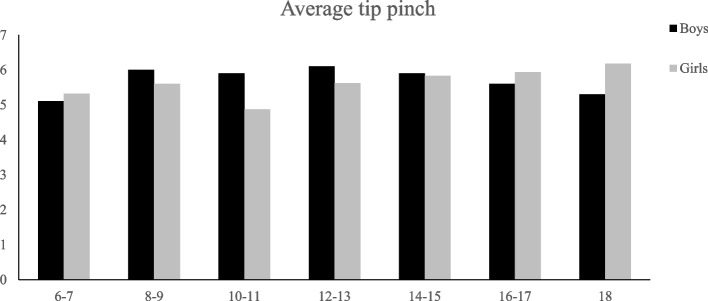
Average Performance of girls and boys on tip pinch strength

**Fig. 4:**
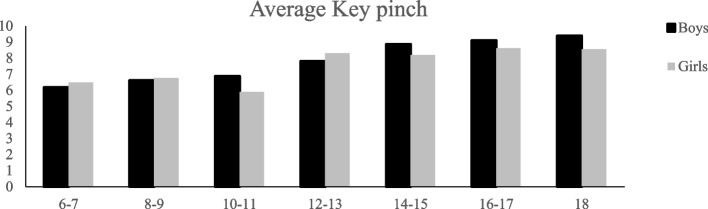
Average Performance of girls and boys on key pinch strength

**Fig. 5:**
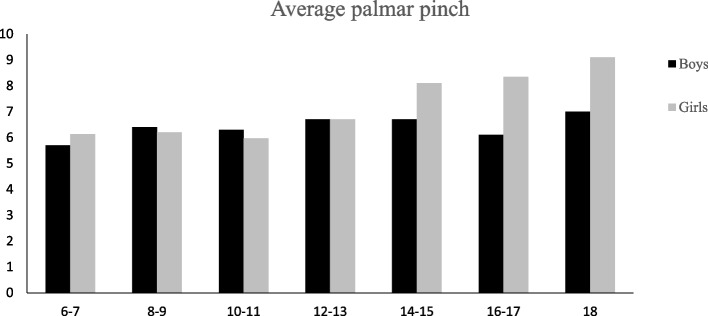
Average Performance of girls and boys on palmar pinch strength

There were 616 participants included in this study (318 boys and 298 girls) between the age of 6 years and 18 years old. Participants were stratified into 5 chronological age groups of 6–7 years, 8–9, 10–11, 12–13, 14–15, 16–17, and 18 years. Characteristics of participants are presented in Table 1. Average performance, SDs, and ranges of grip and pinch strength measures are shown in Tables 2 and 3, and Figs. 2, 3, 4 and 5. In terms of gender differences, boys had significantly higher grip strength than girls in all age groups (*P* < 0.001). However, there was nn significant difference between noys and girls for the other variables (Tables 4).

**Table 1:** Characteristics of participants: age, sex, and hand dominance (*n* = 616)

Age	No. of Boys	Dominance		No. of girls	Dominance	
		Right	Left		Right	Left
6-7	75	69	6	51	48	3
8-9	53	46	7	54	47	7
10-11	42	40	2	45	44	1
12-13	60	53	7	42	38	4
14-15	46	43	3	54	51	3
16-17	35	33	2	40	37	3
18	7	7	0	12	12	0
**Total**	318	291 (91%)	27 (8.4%)	298	277 (93%)	21 (7%)

**Table 2:** Average performance of boys on grip strength, tip pinch, key pinch, and palmar pinch (*n* = 318)

Age	Hand	Grip strength (kg)			Tip Pinch			Key Pinch			Palmar Pinch		
		Mean	SD	Range	Mean	SD	Range	Mean	SD	Range	Mean	SD	Range
6-7	D	9.45	3.6	4-23.8	5.11	3.45	0.83-15.6	6.18	3.07	1.6- 14	5.7	3.1	0.5-12.3
	ND	8.81	3	4.33-21.3	5.1	3.6	0.5-18.33	5.58	2.57	1-10.1	5.5	3.2	0.5-13
8-9	D	10.9	3.4	3.2-17.6	6	4.1	0.5-16.6	6.61	3.4	0.83-16	6.4	3.6	0.5-14.6
	ND	10.4	3.4	3.16-16.2	6.18	3.8	0.5-14.3	6.31	3.21	0.5-15.2	6.17	3.59	0.5-15.4
10-11	D	13.4	5.2	4.6-25.5	5.9	4.3	1-19.5	6.87	2.53	2.66- 12	6.3	3.7	1.8-13.8
	ND	13.1	4.8	4.5-23.9	5.8	4.96	1.2-23	6.88	3.13	3.16-16	6	3.3	1.6-12.3
12-13	D	17.9	7.4	7.4-37.8	6.1	4.1	1.33-17.3	7.8	3.25	2.5-16.6	6.7	3.8	1-14.4
	ND	16.7	7.3	6.3-34.9	5.7	3.8	1-15.2	7.39	3.16	2.33-15.1	6.2	3.4	1-13.1
14-15	D	23.17	10.1	5.8-43.2	5.9	2.5	1.16-12.3	8.87	3.39	3.3-23.4	6.7	3.4	0.83-17.3
	ND	21.6	9.4	5.7-37.2	5.4	2.6	1-12.5	8.58	3.27	2.16-20.16	6.3	3.22	1-15.8
16-17	D	26.2	7.9	7.6-47.2	5.6	2.2	1.66-11	9.1	2.66	4.33-19.5	6.1	2.4	1.8-11.5
	ND	25.5	7.5	7.4-38.1	5.4	2.1	1.66-11.9	8.72	3.29	3.66-23.1	5.9	2.7	1.66-13
18	D	30.1	9.2	11.2-43.1	5.3	1.17	4.33-7.6	9.39	1.41	6.83-11.6	7	1.9	3.6-10
18	ND	26.9	7.75	11-38.3	5.31	1.4	3.8-8.4	8.84	1.35	6.66-10.8	6.7	2.1	3.33-9.9

*D* Dominant hand, *ND* Non-dominant hand

**Table 3:** Average performance of girls on grip strength, tip pinch, key pinch, and palmar pinch (*n* = 298)

Age	Hand	Grip strength (kg)			Tip Pinch			Key Pinch			Palmar Pinch		
		Mean	SD	Range	Mean	SD	Range	Mean	SD	Range	Mean	SD	Range
6-7	D	10.5	5.8	1-24.1	5.32	2.93	0.5-11	6.49	3.45	1-11.9	6.13	2.91	0.66-10.26
	ND	9.56	4.63	1-20.4	4.97	2.23	0.66-10.3	5.85	3.17	1-15.1	5.73	2.87	0.5-10.23
8-9	D	10.5	3.45	3.93-17.9	5.60	3.46	0.5-23.16	6.76	2.82	1.16-12	6.2	3.1	0.5-12.6
	ND	10.17	3.50	3.50-20.3	5.23	3.05	0.5-18.1	6.51	3.14	1.3-15.16	5.75	2.79	0.5-12.3
10-11	D	12.2	4.46	3.83-21.6	4.87	2.88	0.5-12	5.89	2.73	0.66-15	5.97	3.41	1.16-13.2
	ND	11.67	4.7	2.83-28.5	4.80	3.16	0.5-14.3	5.96	2.76	0.6-14	5.39	3.1	0.5-13
12-13	D	14.8	6.43	3.6-28.3	5.62	3.10	0.5-13.4	8.3	3.81	1.83-16.93	6.7	3.67	0.5-13.43
	ND	13.9	5.47	3.46-24.1	5.14	2.83	0.5-10.16	7.44	3.56	1.8-15.23	6.12	3.53	0.66-13.8
14-15	D	14.8	6.55	6.5-26.1	5.83	3.67	0.5-17.3	8.2	3.34	2-15.2	8.1	5.81	0.83-26.5
	ND	13.8	5.9	5.83-23.9	5.90	4.51	0.5-21.66	7.21	3.73	1.5-15.16	7.10	5.36	0.33-23.5
16-17	D	14.7	6.90	6.1-27.8	5.94	2.89	0.66-11.5	8.61	3.44	2.16-16.3	8.35	4.54	0.5-16.66
	ND	13.2	6.22	6.13-26.4	5.30	2.88	0.5-11.66	7.92	3.77	1-16.66	7.3	4.68	0.16-17.3
18	D	17.3	9.22	7-36.1	6.18	3.43	2.5-15.1	8.55	3.1	4-15.1	9.1	5.92	2.6-22
18	ND	15.1	7.96	6.2-32	5.20	2.18	1.83-10.16	7.5	2.68	4.16-13.5	8.25	5.40	1.83-17.33

*D* Dominant hand, *ND* Non-dominant hand

**Table 4:** Differences in grip and pinch strength measurements between boys and girls (*n* = 616)

Age	Sex	Grip strength (kg)	Tip pinch strength (kg)	Key pinch strength (kg)	Palmar pinch strength (kg)
6 (*n* = 28)	Boys (*n* = 14)	8.5 ± 6.1	5.7 ± 4.3	4.6 ± 1.5	5.2 ± 2.6
	Girls (*n* = 14)	8.5 ± 5	4.3 ± 2.5	5.1 ± 3.4	6.2 ± 3.7
7 (*n* = 98)	Boys (*n* = 61)	18.6 ± 10.5	4.9 ± 2.6	7.6 ± 4	5.7 ± 3.2
	Girls (*n* = 37)	13.3 ± 5.8	4.4 ± 2.4	5.5 ± 2.1	5.9 ± 3.8
8 (*n* = 45)	Boys (*n* = 22)	15.8 ± 9.6	5.5 ± 2.49	7.3 ± 2.7	6.5 ± 3.2
	Girls (*n* = 23)	13.5 ± 6.5	5.8 ± 3.3	8.04 ± 4.7	6.5 ± 3.7
9 (*n* = 62)	Boys (*n* = 31)	18.7 ± 9.7	5.9 ± 3.04	7.8 ± 2.4	6.6 ± 2.9
	Girls (*n* = 31)	12.5 ± 6.30	5.3 ± 2.02	7.4 ± 2.9	6.9 ± 3.1
10 (*n* = 34)	Boys (*n* = 15)	15.1 ± 9.8	6.2 ± 5.2	7.6 ± 2.9	6.5 ± 3.8
	Girls (*n* = 19)	12.7 ± 5.1	5.3 ± 3.8	6.7 ± 3.5	5.9 ± 3.8
11 (*n* = 53)	Boys (*n* = 27)	12.4 ± 4.8	6.1 ± 4.1	7.2 ± 2.9	6.7 ± 3.4
	Girls (*n* = 26)	12.9 ± 5.7	5.6 ± 3.18	6.7 ± 3.9	7.03 ± 4.1
12 (*n* = 53)	Boys (*n* = 31)	17.4 ± 6.6	6.5 ± 4.3	7.7 ± 3.7	6.7 ± 4.2
	Girls (*n* = 22)	12.0 ± 6.5	5.7 ± 4.6	6.9 ± 2.9	6.2 ± 3.4
13 (*n* = 49)	Boys (*n* = 29)	17.8 ± 9.5	5.9 ± 3.1	8.1 ± 3.1	6.9 ± 3.5
	Girls (*n* = 20)	13.4 ± 6.4	6.3 ± 3.7	8.2 ± 3.7	7.3 ± 4
14 (*n* = 49)	Boys (*n* = 23)	16.6 ± 8	4.9 ± 2.4	8.5 ± 3.6	6.1 ± 2.9
	Girls (*n* = 26)	12.5 ± 6.7	5.7 ± 2.6	7.9 ± 2.9	7.3 ± 3.9
15 (*n* = 51)	Boys (*n* = 23)	14.5 ± 8.2	5.6 ± 2.9	7.7 ± 3.1	6.5 ± 3.4
	Girls (*n* = 28)	13.2 ± 5.01	5.9 ± 3.7	7.6 ± 2.1	7.4 ± 5.6
16 (*n* = 44)	Boys (*n* = 19)	14.9 ± 8.8	6.3 ± 3.6	8.3 ± 3.7	6.4 ± 3.4
	Girls (*n* = 25)	12.4 ± 6.2	5.8 ± 2.8	8.1 ± 3.8	7.3 ± 4.3
17 (*n* = 31)	Boys (*n* = 16)	16.6 ± 8.1	6.6 ± 3.6	8.1 ± 2.6	7.6 ± 3.7
	Girls (*n* = 15)	12.8 ± 4.6	4.8 ± 2.5	7.4 ± 3.6	6.3 ± 3.4
18 (*n* = 19)	Boys (*n* = 7)	14.5 ± 6.8	5.4 ± 1.9	7.6 ± 2.5	6.6 ± 2.9
	Girls (*n* = 12)	14.5 ± 6.8	5.9 ± 2.5	8.5 ± 2.9	7.8 ± 3.6
*P*value*		*P* < 0.001	*P* = 0.589	*P* = 0.083	*P* = 0.383

Values are given as mean ± standard deviation. * *P* value for the difference between boys and girls

## Discussion

This study established a normative data of hand grip strength and pinch strength for Saudi healthy children aged 6 to 18 years old. To our knowledge this is the first published nation-wide study that accounted for socioeconomical differences by including different regions in Saudi Arabia (central and southern). The results showed an overall trend of increasing hand grip strength and pinch strength with age regardless of hand preference, which were consistent with previous findings [4, 11, 16, 17, 24, 26]. This pattern of incresed strength might be explained by the physiological changes and development of arm and forearm muscle strength in both genders with age. In 2021, Rostamzadeh et al., reported hand grip strength norms for Iranian children/adolescents aged 7 to 18 years old [24]. The hand grip strength trend with age was not significantly different compared to our findings. However, younger Iranian children (age 7 to 10 years) showed weaker hand grip strength compared to our sample, while older Iranian children (older than 11 years) presented with stronger hand grip strength compared to Saudi counterparts.

Boys have presented with higher hand grip strength and pinch grip compared to girls across different age groups, however a variation in hand grip strength was more evident in boys compared to girls. This variation could be attributed to underrepresentation of sample size in some age groups. Moreover, the physical activity level, type of exercise practiced by boys, and lack of physical education classes in girls’ schools might play a vital role in this gender difference. Compared to previously published studies, similar hand grip strength and pinch strength could be observed in the younger age group (6-11 years old). However, this similarity with other studies in older age group was not clear [4, 17, 24]. Other studies have reported that puberty played a role in accelerating the hand grip strength and pinch strength in boys compared to girls around the age of 10 to 13 years old [27, 28].

In the current study, we used procedures similar to that reported in previous studies for hand grip and pinch strength [4, 11, 24, 29]. Although every possible effort has been made, the level of agreement might be affected across populations [4, 11, 24, 29]. Comparing our sample with already existing data without any consideration of the type of dynamometer used and the protocol procedure followed is challenging. Another reason for the difficulty of comapriing our results to previous reports is the lack of studies in the regions such as the Middle East and Saudi Arabia. For example, comparing children from Saudi Arabia might not be accurate when they compared to western countries such as the United States due to physical and environmental differences. Future work is needed from areas with similar physical and environmental factors. Finally, future reseach should consider utilizing reliable and valid dynamometers following a standardized procedure of data collection to overcome such variability.

Understanding the normative values of hand parameters will facilitate tracking performance in rehabilitation sittings. It has been suggested to include race and ethnicity in hand function research because of the demographics influence on clinical practice [4]. As mentioned, there is lacking of evidence of norms values in Saudi pediatric population in which the findings of this paper may contribute to the clinical decision making for Saudi children. The data collection was initiated during the Covid-19 pandemic and all children required to attend classes in online format. This might be the reason for lower values of all hand parameters compared to a meta-analysis studies in which the high demand for hand function might increase fatigue and weakness on hands [4]. In Saudi Arabia, smartphones or tablets have been used for online classes during pnadmeic. Excessive use of smartphones or tablets have been linked to a decrease in grip and pinch strength [30]. Therefore, our results might be affected by online class. We recommend future research to include devices usage duration as a factor of normative values of hand parameters for children. However, without established data of normative values using inexpensive objective tools for hand function, there will be difficulties to understand progress after hand surgeries for this population.

This study has some limitations that should be considered. It is unknown whether other factors might affect pinch and grip strength such as nutritional status, exercise profile, sports profile, and socioeconomic level. These factors should be considered in future reseach to identify the relationship between demographics and socioeconomic factors and pinch strength in children. Another limitation of the current study is using different types of dynamometers that might have an impact on the results due to different measurement errors of the tools. This study, on the other hand, trumps earlier studies by presenting data from both urban and rural areas across the country. Although this research included data from a variety of chronological age groups, some chronological age groups were underrepresented in the sample.

## Conclusion

The present study establishes reference values for grip and pinch strength in healthy Saudi pediatric and adolescent population, using boys and girls aged 6 to 18. The findings followed a trend that had previously conducted similar research in other nations. However, Saudi boys and girls were lower in grip and pinch strength than those of their western counterparts. Numerous factors, such as hormonal and dietary changes, sociodemographic and anthropometric differences, as well as COVID-19, may be at play in the differences. The outcomes of this study also demonstrated that gender, age, and hand preference can all have an impact on how strong a handgrip develops. Boys of all ages had greater grip and pinch strength than girls. The normative data on hand function may aid in measuring the effectiveness of rehabilitation programs. A future reseach is needed to identify the relationship between demographics and socioeconomic factors and pinch strength in children in Saudi Arabia.

## Data Availability

Data used in the study is available from the corresponding author on reasonable request.
